# Generation of Monoclonal Antibodies against Dengue Virus Type 4 and Identification of Enhancing Epitopes on Envelope Protein

**DOI:** 10.1371/journal.pone.0136328

**Published:** 2015-08-26

**Authors:** Chung-Tao Tang, Mei-Ying Liao, Chien-Yu Chiu, Wen-Fan Shen, Chiung-Yi Chiu, Ping-Chang Cheng, Gwong-Jen J. Chang, Han-Chung Wu

**Affiliations:** 1 Institute of Cellular and Organismic Biology, Academia Sinica, Taipei, Taiwan; 2 Arbovirus Diseases Branch, Division of Vector-Borne Infectious Diseases, Centers for Disease Control and Prevention, Public Health Service, United States Department of Health and Human Services, Fort Collins, Colorado, United States of America; New York State Dept. Health, UNITED STATES

## Abstract

The four serotypes of dengue virus (DENV1-4) pose a serious threat to global health. Cross-reactive and non-neutralizing antibodies enhance viral infection, thereby exacerbating the disease via antibody-dependent enhancement (ADE). Studying the epitopes targeted by these enhancing antibodies would improve the immune responses against DENV infection. In order to investigate the roles of antibodies in the pathogenesis of dengue, we generated a panel of 16 new monoclonal antibodies (mAbs) against DENV4. Using plaque reduction neutralization test (PRNT), we examined the neutralizing activity of these mAbs. Furthermore, we used the *in vitro* and *in vivo* ADE assay to evaluate the enhancement of DENV infection by mAbs. The results indicate that the cross-reactive and poorly neutralizing mAbs, DD11-4 and DD18-5, strongly enhance DENV1-4 infection of K562 cells and increase mortality in AG129 mice. The epitope residues of these enhancing mAbs were identified using virus-like particle (VLP) mutants. W212 and E26 are the epitope residues of DD11-4 and DD18-5, respectively. In conclusion, we generated and characterized 16 new mAbs against DENV4. DD11-4 and D18-5 possessed non-neutralizing activities and enhanced viral infection. Moreover, we identified the epitope residues of enhancing mAbs on envelope protein. These results may provide useful information for development of safe dengue vaccine.

## Introduction

There are an estimated 390 million dengue infections every year, mostly in tropical and subtropical areas [1]. Dengue infection can cause asymptomatic dengue fever (DF), as well as more life-threatening illness, such as dengue hemorrhagic fever (DHF) and dengue shock syndrome (DSS) [2]. Although initial infection with DENV provides immunity against the same serotype, subsequent infection by other serotypes can result in a more severe disease [3, 4]. The presence of non-neutralizing and sub-neutralizing antibodies bound to DENV exacerbates the disease by binding to the Fcγ receptors (FcγR) of cells. This hypothetical process is termed antibody-dependent enhancement (ADE) [3, 5]. At the time of writing, there is no approved vaccine or therapy that can alleviate the symptoms of dengue infection [6].

DENV, which consists of four closely related serotypes (DENV1-4), is a member of the *Flavivirus* genus within the *Flaviviridae* family [7]. The genome of DENV is a positive-strand RNA of about 11 kb in length. The viral RNA is translated into a single polyprotein that is cleaved by cellular and viral proteases into three structural proteins [capsid (C), premembrane (prM), and envelope (E) proteins] and seven non-structural proteins (NS1, NS2A, NS2B, NS3, NS4A, NS4B, and NS5 proteins) [8, 9]. After replication, the virus is assembled and subsequently transported to the Golgi. In the acidic environment of the trans-Golgi network (TGN), the prM protein is cleaved by furin to generate mature virions [10]. Co-expression of prM and E proteins can produce recombinant virus-like particles (VLPs), which are similar in structure and antigenicity to infectious virus particles, and have been used broadly in epitope mapping, diagnosis, and development of vaccines [11, 12]. In addition, NS1 protein, the secreted nonstructural glycoprotein, also plays a critical role in pathogenesis of DENV infection. Antibodies against NS1 can bind to endothelial cells and cause apoptosis [13, 14].

The E protein is required for viral attachment to cell surface receptor(s), fusion with endosomal membranes, and entry into target cells. Thus, the E protein is regarded as an important target for neutralizing DENV [15–18]. In the mature virion, the E protein forms 90 homodimers on the surface of the virus particle [19]. Crystallographic analysis of E protein has shown that it is divided into three distinct domains: domain I (EDI), domain II (EDII), and domain III (EDIII) [20]. EDI, which links EDII with EDIII, is organized as an eight-stranded central β–barrel structure, and is involved in conformational changes. EDII is an elongated dimerization domain, which contains a fusion loop at the tip [21]. EDIII is an immunoglobulin-like region, which is thought to be the binding site of the cell receptor on the target cell [22]. The mAbs against EDIII are largely serotype-specific, and block virus infection [18, 23, 24]. In dengue pathogenesis, cross-reactive and non-neutralizing antibodies against E proteins from primary infection are highly potent at enhancing viral infection through ADE during secondary infection [25, 26]. Analyses of the antigenic characteristics of cross-reactive and weakly-neutralizing antibodies have elucidated their binding specificities and functional activities.

While previous studies have focused on the roles of epitopes in the neutralization of DENV [12, 15, 18, 23], here we considered the epitopes recognized by cross-reactive and weakly-neutralizing antibodies that are involved in enhancing viral infection by ADE. In this study, we generated a panel of 16 mAbs against DENV4; we then characterized their virus specificities and binding regions by enzyme-linked immunosorbent assay (ELISA), immunofluorescence assay (IFA), and Western blotting (WB). Using plaque reduction neutralization test (PRNT), the *in vitro* ADE assay, and *in vivo* enhancement of mortality in AG129 mice, we found that the cross-reactive DD11-4 and DD18-5 mAbs had poorly neutralizing activities and enhanced DENV infection. To further identify the epitope residues of enhancing DD11-4 and DD18-5, we used bioinformatic analysis, site-directed mutagenesis, and binding assays with VLP mutants. We identified that W212 and E26 are the epitope residues of enhancing DD11-4 and DD18-5, respectively. The identification of these epitope residues may provide valuable information for improving immune responses against DENV infection.

## Materials and Methods

### Ethics Statement

The animal care were carried out in strict accordance with the guidelines of the Academia Sinica Institutional Animal Care and Use Committee. The protocol was approved by the Committee on the Ethics of Animal Experiments of Academia Sinica (Permit Number: 11-04-166). For mouse experiments, the mice were monitored every day, and used human endpoints by judgment of the mouse weight loss (>20% of body weight) or assessment of mouse activity (hunching, stationary, ruffled fur, and poor grooming). At the end of the experiments, the mice were sacrificed with CO_2_ asphyxiation. All efforts were made to minimize suffering.

### DENV and cells

Four dengue viruses, DENV1 Hawaii, DENV2 16681, DENV3 H87, and DENV4 H241, were prepared as previously described [27]. All viral strains were used to infect mosquito C6/36 cells (ATCC CRL-1660) with growth media consisting of 50% Mitsumashi and Maramorsch insect medium (Sigma) plus 50% Dulbecco’s modified Eagle’s minimal essential medium (DMEM; Gibco), as previously described [18]. The DENV-infected C6/36 cells were incubated at 28°C for 7 to 9 days. The viruses were harvested from supernatant and titrated in a baby hamster kidney fibroblast cell line, BHK-21 (ATCC CCL-10), by plaque assay. The plaque-forming units (pfu) were counted. BHK-21 cells were grown in minimal essential medium (MEM) containing 10% heat-inactivated fetal bovine serum (FBS).

### Generation of mAbs against DENV4

The anti-DENV4 monoclonal antibodies (mAbs) were generated by hybridomas, as previously described [28]. Briefly, the DENV4 virions were emulsified in Freund’s adjuvant (Sigma), and intraperitoneally (i.p.) injected into female BALB/c mice four times at 3-week intervals. After the last immunization, splenocytes were collected and fused with myeloma cells using 50% (v/v) polyethylene glycol (Gibco). The fused cells were resuspended in DMEM containing 15% FBS, hypoxanthine-aminopterin-thymidine medium, and hybridoma cloning factor (ICN, Aurora, OH). The culture supernatants from hybridoma cells were screened by cellular ELISA to characterize the mAbs bound to DENV-infected C6/36 cells, as previously described [18]. In brief, the C6/36 cells were infected with DENV1-4. After fixation and permeabilization, the supernatants from hybridoma colonies were incubated with infected cells prior to assessment. Selected clones were subcloned by limiting dilution, isotyped (SouthernBiotech), and purified using protein G Sepharose 4B gels (GE Healthcare).

### Screening of mAbs against DENV by ELISA

ELISA plates were coated with a 1:800 dilution of polyclonal rabbit anti-DENV hyper-immune sera for 24 hours at 4°C. After blocking, the plates were incubated with diluted viral supernatants of DENV1-4 for 1 hour at room temperature (RT). DENV was detected with mAbs (1 μg/ml) for 2 hours at RT. The plates were washed three times with phosphate-buffered saline containing 0.1% (w/v) Tween 20 (PBST_0.1_), and then incubated with horseradish peroxidase (HRP)-conjugated anti-mouse immunoglobulin G (IgG; Jackson ImmunoResearch Laboratories). After washing, *o*-phenylenediamine dihydrochloride (OPD; Sigma) was added into the plates. The reaction was stopped with 3N HCl, and analyzed using a microplate reader at 490 nm. The mean optical density (OD) of mock supernatants plus three standard deviations was used to determine the cutoff value for this assay.

### Immunofluorescence assay (IFA)

BHK-21 cells were cultured on slides in a 24-well plate overnight. The cells were infected with DENV1-4 at multiplicity of infection (MOI) of 1–10. After 48 hours, the infected cells were fixed with 1:1 methanol/acetone at -20°C for 10 minutes. The slides were washed with PBS, and then blocked with 1% bovine serum albumin (BSA) in PBS at RT for 30 minutes. Next, the infected cells were incubated with mAbs at RT for 1 hour. After washing with PBST_0.1_, the slides were stained with fluorescent-isothiocyanate (FITC)-labeled goat anti-mouse IgG (Jackson ImmunoResearch Laboratories) and 4’,6-diamidino-2-phenylindole (DAPI; Invitrogen) at RT for 1 hour. After washing, the slides were examined under a fluorescent microscope.

### Expression of recombinant EDI-II and EDIII proteins of DENV4

The EDI-II of DENV4, comprising amino acids 1–296 of the E protein, was cloned into the pET21a vector (Merck). The EDIII of DENV4, comprising amino acids 295–397 of the E protein, was also cloned into the pET21a vector. The pET-21a/rD4EDI-II and pET-21a/rD4EDIII plasmids were transformed and expressed in *Escherichia coli* BL21 (DE3). The colonies were inoculated and grown in LB media containing ampicillin at 37°C overnight. The cultures were then diluted and induced with 1 mM isopropylthiogalactoside (IPTG) at 37°C for 4 hours. After collection, the lysates from bacterial cells were sonicated on ice and centrifuged at 10,000g for 30 minutes. Finally, the recombinant EDI-II and EDIII proteins of DENV4 were harvested.

### Western blot analysis

C6/36 cells were infected at an MOI of 0.1 with DENV1-4, and infected cells were then harvested and lysed. The cell lysates were mixed with sample buffer (Bio-Rad Laboratories). Next, protein samples were separated by non-reducing sodium dodecyl sulfate-polyacrylamide gel electrophoresis (SDS-PAGE) and transferred to a nitrocellulose membrane (Hybond-C Extra; Amersham Biosciences). The membrane was blocked with 5% skimmed milk in PBS and detected with different mAbs. After washing, the membrane was incubated with HRP-conjugated goat anti-mouse IgG (Jackson ImmunoResearch Laboratories) and developed with chemiluminescence reagents (ECL; Amersham). The recombinant proteins were separated by reducing SDS-PAGE. The transfer and staining conditions were as above.

### Plaque reduction neutralization test (PRNT)

For maintaining the viral activities, the diluted mAbs were incubated with DENV1-4 at 4°C for 1 hour, as previously described [18]. The mixtures were added to infect BHK-21 cells, with each experiment performed in triplicate. After adsorption of viruses at 37°C for 2 hours, 2 ml of MEM containg 2% FBS and 1% methyl-cellulose were added to each well. After 4 days, the cells were fixed with 3.7% formaldehyde for 1 hour and stained with 0.5% crystal violet for 20 mins. The plates were washed with tap water, and the viral plaques that formed were counted. Plaque reduction was calculated as follows: Inhibition percentage = 100 × [1 - (plaque number incubated with mAb/plaque number without mAb)]. The titer is expressed in Table 1 as the lowest concentration of mAbs that resulted in a ≧50% plaque reduction (PRNT_50_).

**Table 1:** Characterization of mAb activities against DENV4 by ELISA, IFA, WB, and PRNT_50_.

mAbs	Isotyping	Class^a^	ELISA^b^				IFA^c^				WB^d^				Specificity^e^	DENV4 PRNT_50_ (μg/ml)^f^
			D1	D2	D3	D4	D1	D2	D3	D4	D1	D2	D3	D4		
DD1-4	IgG2a, κ	Type	-	-	-	+	-	-	-	+	-	-	-	+	EDI-II	>40
DD3-7	IgG1, κ	Type	-	-	-	+	-	-	-	+	-	-	-	+	NS1	n.d.
DD4-3	IgG2a, κ	Type	-	-	-	+	-	-	-	+	-	-	-	+	E	>40
DD5-1	IgG1, κ	Type	-	-	-	+	-	-	-	+	-	-	-	+	E	>40
DD7-8	IgG2a, κ	Subcomplex	+	-	-	+	+	-	-	+	+	-	-	+	NS1	n.d.
DD9-4	IgG2a, κ	Type	-	-	-	+	-	-	-	+	-	-	-	+	E	>40
DD11-4	IgG1, κ	Complex	+	+	+	+	+	+	+	+	+	+	+	+	EDI-II	>40
DD13-4	IgG1, κ	Type	-	-	-	+	-	-	-	+	-	-	-	+	EDI-II	>40
DD14-1	IgG2a, κ	Subcomplex	-	-	+	+	-	-	+	+	-	-	+	+	EDI-II	>40
DD15-2	IgG2b, κ	Subcomplex	-	-	+	+	-	-	+	+	-	-	+	+	E	>40
DD17-4	IgG2a, κ	Type	-	-	-	+	-	-	-	+	-	-	-	+	EDIII	>40
DD18-5	IgG2a, κ	Complex	+	+	+	+	+	+	+	+	+	+	+	+	EDI-II	≧20
DD27-8	IgG2a, κ	Type	-	-	-	+	-	-	-	+	-	-	-	+	EDIII	>40
DD30-4	IgG2a, κ	Type	-	-	-	+	-	-	-	+	-	-	-	+	EDI-II	>40
DD31-3	IgG1, κ	Type	-	-	-	+	-	-	-	+	-	-	-	+	EDI-II	>40
DD33-2	IgG1, κ	Subcomplex	-	+	-	+	-	+	-	+	-	+	-	+	NS1	n.d.

^a^ The classes of mAbs include complex-reactive, subcomplex-reactive, and type-specific. The results were determined by ELISA, IFA, and WB.

^b^ Plates coated with rabbit hyper-immune sera against DENV1-4 were used to test the reactivity of mAbs. (+) The OD values above cut off value were positive (+), and those below were negative (-).

^c^ The IFA results using DENV1-4-infected BHK-21 cells were confirmed as described in the Methods.

^d^ Western blotting (WB) using cell lysates from DENV1-4-infected C6/36 cells was performed as described in the Methods.

^e^ The specificities of mAbs were determined using lysates of virus-infected cells. The binding domains of mAbs were identified by recombinant proteins. E: envelope protein; EDI-II, recombinant domain I-II of E protein; EDIII, recombinant domain III of E protein; NS1, nonstructural protein 1.

^f^ The PRNT_50_ values are shown as the lowest concentration of mAbs that reduced ≧50% of plaques. (n.d.) not determined.

### *In vitro* ADE assay of DENV with mAbs

Dilutions of the various mAbs were mixed with DENV1 (MOI = 1), DENV2 (MOI = 1), DENV3 (MOI = 5), or DENV4 (MOI = 1) for 1 hour at 4°C; the mixtures were then used to infect K562 cells. After 3 days, the cells were fixed with 3.7% formaldehyde, permeabilized in 2% FBS PBS containing 0.1% saponin, and stained with 4G2. After washing, the cells were incubated with R-phycoerythrin (RPE)-conjugated goat anti-mouse IgG (Jackson ImmunoResearch Laboratories), and analyzed by flow cytometry. Normal mouse IgG (NMIgG) was used as a negative control. In addition, DENV2 S221 (MOI = 5) was incubated with diluted mAbs for 1 hour at 4°C. Subsequently, the mixtures were added to infect K562 cells. After 3 days, the cells were fixed, permeabilized, and stained with 4G2. After washing, the cells were incubated with the RPE-conjugated goat anti-mouse IgG (Jackson ImmunoResearch Laboratories), and analyzed by flow cytometry.

### Measurement of *in vivo* ADE with mAbs in AG129 mice

Type I and II interferon receptor-deficient mice (AG129) at 5- to 6-week-old were purchased from B&K Universal. The AG129 mice were administered intraperitoneally (i.p.) with 5 μg mAbs in 200 μl PBS on days -1 and 1. On day 0 of infection, mice were intravenously (i.v.) inoculated with 1 × 10^5^ pfu of the mouse-adapted DENV2 S221 (obtained from S. Shresta) [29], in 100 μl PBS. NMIgG was used as a control. The survival rates were recorded for 30 days.

### Identification of epitope residues by cells expressing wild-type and mutant DENV1-4 prM/E proteins

The pCBD1, pCBD2-2J-2-9-1, pCBD3, and pCBD4 constructs express prM/E proteins of DENV1-4 [30]. Site-directed PCR mutagenesis of the selected amino acid residue was performed. After mutagenesis, the constructs were sequenced to confirm that there were no unintended mutations at other sites. The BHK-21 cells were transfected with vector expressing the wild-type or mutant E proteins of DENV1-4 using polyjet *in vitro* DNA transfection reagent (SignGen Laboratories). After 2 days, the cells were collected, fixed, and permeabilized. For staining, cells were incubated with mAbs (DD11-4, DD18-5, or a mixture of mAbs, all at concentrations of 1 μg/ml) at 4°C for 30 min. After washing, cells were incubated with RPE-conjugated goat anti-mouse IgG (Jackson ImmunoResearch Laboratories), and analyzed by flow cytometry. The mixed mAbs against DENV1 EDIII (DA6-7 and DA11-3)[28], DENV2 EDIII (DB32-6, DB25-2, and 3H5)[18], DENV3 EDIII (DC9-6, DC12-3, and DC14-3) (generated by our laboratory), and DENV4 EDIII (DD17-4 and DD27-8) were used as positive controls for the relevant experiments. The binding index of a mAb to a mutant E protein was measured using the following formula: (intensity of the mutant E/intensity of wild-type E [by mAb])/(intensity of mutant E/intensity of wild-type E [by mixed mAbs]) [31].

### Statistical analysis

For the *in vitro* ADE assay of mAbs, Student’s *t* tests were used to determine the significant differences and calculate *P* values (**P<0.01, ***P<0.001). Survival rates were expressed using Kaplan-Meier survival curves, and statistical analyses were performed using GraphPad Prism 5.

## Results

### Generation and characterization of mAbs against DENV

**Fig 1:**
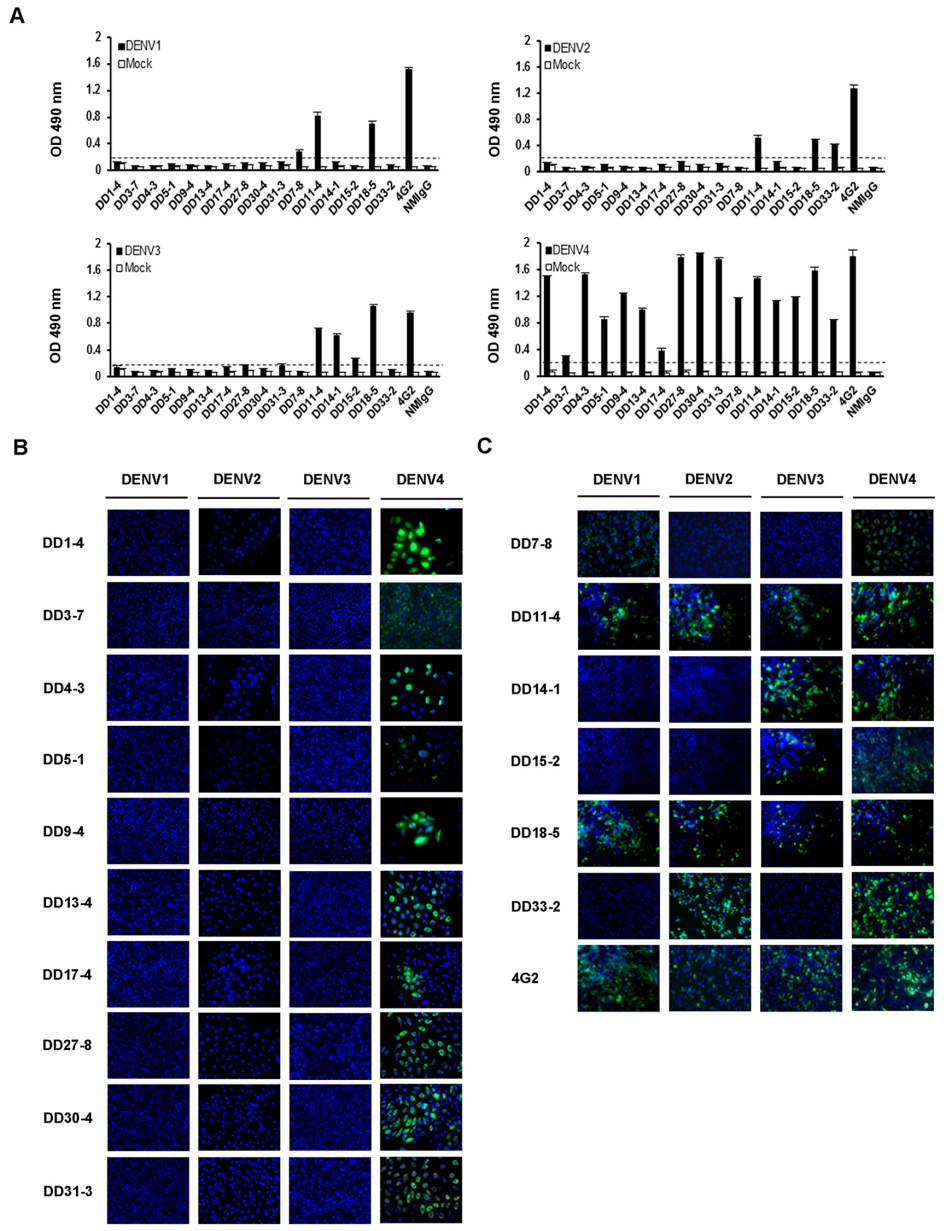
Characterization of mAbs against DENV1-4. (A) ELISA plates were coated with 1:800 dilutions of polyclonal rabbit anti-DENV hyper-immune sera at 4°C for 24 hours. After blocking, DENV1-4 viral supernatants were added, which were then detected by incubation with 1 μg/ml mAbs at RT for 2 hours. Next, the plates were incubated with HRP-conjugated goat anti-mouse IgG, and developed using OPD. The OD at a wavelength of 490 nm was measured. The cutoff values are represented by dotted lines. (B and C) BHK-21 cells were infected with DENV1-4, respectively. After 2 days, the infected cells were detected with mAbs. DD1-4, DD3-7, DD4-3, DD5-1, DD9-4, DD13-4, DD17-4, DD27-8, DD30-4, and DD31-3 are DENV4 serotype-specific mAbs. DD7-8, DD11-4, DD14-1, DD15-2, DD18-5, and DD33-2 are cross-reactive mAbs. 4G2 is the positive control.

A total of 16 mAbs against DENV were generated in this study. The reactivity of these mAbs with DENV1-4 was determined using ELISA (Fig 1A). In order to further confirm the specificity of these mAbs, BHK-21 cells were infected with DENV1-4, respectively. After 2 days, the infected cells were detected with mAbs by IFA (Fig 1B and 1C). Ten of the mAbs are serotype-specific antibodies against DENV4; these mAbs are as follows: DD1-4, DD3-7, DD4-3, DD5-1, DD9-4, DD13-4, DD17-4, DD27-8, DD30-4, and DD31-3 (Fig 1B). In addition, cells infected with DENV4 or the other serotypes of DENV were recognized by DD7-8, DD11-4, DD14-1, DD15-2, DD18-5, and DD33-2. These six mAbs were cross-reactive (Fig 1C). 4G2 against DENV1-4 was used as a positive control (Fig 1C).

### Determination of binding regions of mAbs in DENV

**Fig 2:**
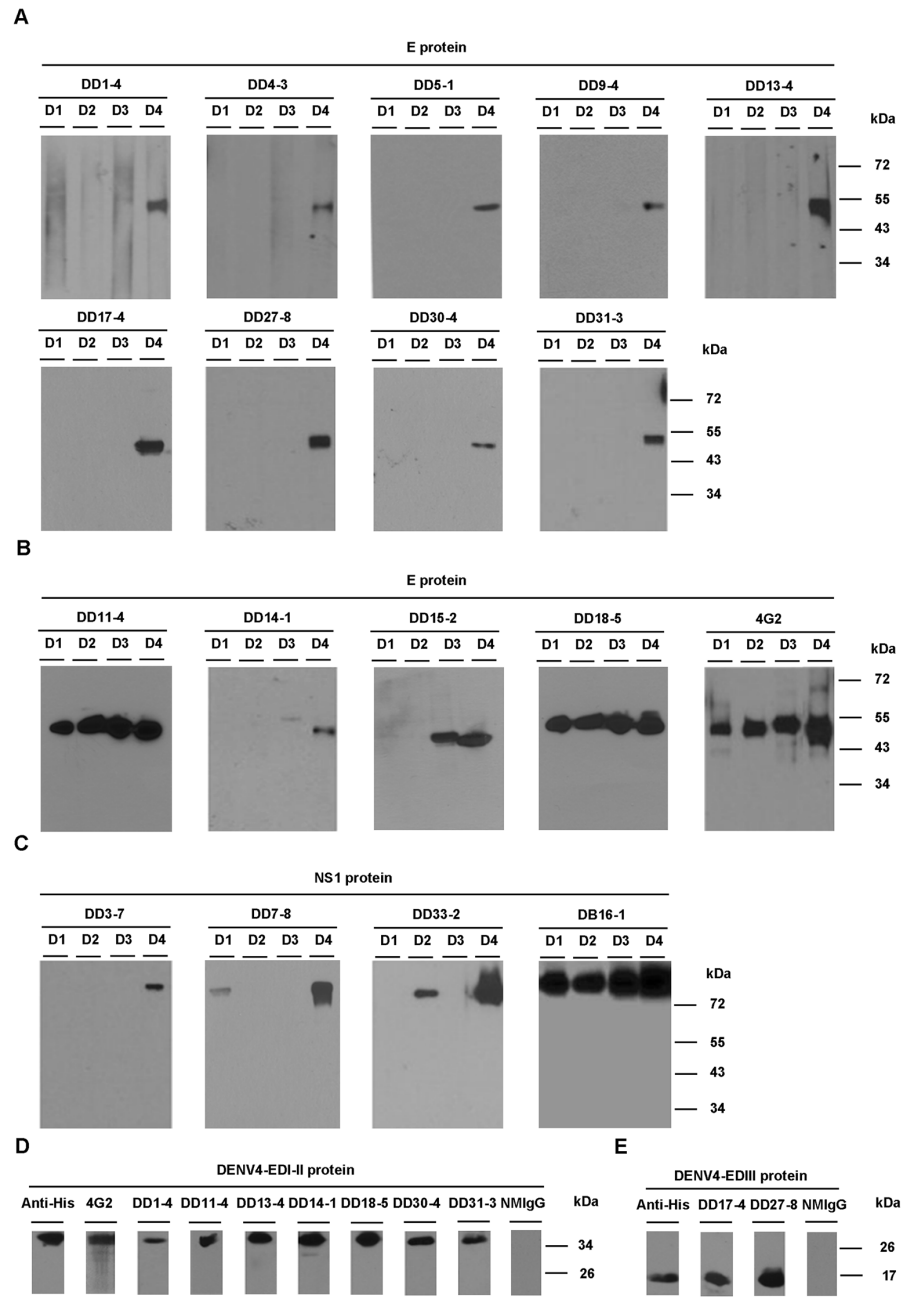
Determination of the binding regions of mAbs by Western blotting. Four serotypes of DENV antigen (D1 to D4) from infected C6/36 cell lysates were analyzed and detected with mAbs by Western blotting. (A) The E proteins (about 53 kDa) of DENV4 were recognized by DD1-4, DD4-3, DD5-1, DD9-4, DD13-4, DD17-4, DD27-8, DD30-4, and DD31-3. (B) The E proteins of DENV3 and DENV4 were recognized by DD14-1 and DD15-2. The E proteins of DENV1-4 were recognized by DD11-4 and DD18-5. (C) The dimeric form of the NS1 proteins (about 75 kDa) was recognized by DD3-7, DD7-8, and DD33-2. DB16-1 was used as a positive control for dimeric NS1 proteins. (D) DD1-4, DD11-4, DD13-4, DD14-1, DD18-5, DD30-4, and DD31-3 recognized the EDI-II protein (about 36 kDa) of DENV4. The 4G2 mAb (which targets the EDI-II protein of DENV4) was used as a positive control. NMIgG was used as a negative control. (E) DD17-4 and DD27-8 recognized the EDIII protein (about 17 kDa) of DENV4. NMIgG was used as a negative control.

We utilized non-reducing SDS-PAGE and Western blotting to identify the target protein of mAbs. The E protein (about 53 kDa) of DENV4 was recognized by the following 9 serotype-specific mAbs: DD1-4, DD4-3, DD5-1, DD9-4, DD13-4, DD17-4, DD27-8, DD30-4, and DD31-3 (Fig 2A). Four mAbs, DD11-4, DD14-1, DD15-2, and DD18-5, recognized the E proteins of DENV4 and the other serotypes of DENV (Fig 2B). Of note, DD11-4 and DD18-5 reacted with DENV1-4 E proteins. DD3-7, DD7-8, and DD33-2 could recognize the dimeric NS1 proteins (about 75 kDa) of DENV (Fig 2C). DB16-1 was used as a positive control for NS1 proteins [14].

Subsequently, we further examined the binding domains of anti-E mAbs using recombinant domain I-II (EDI-II) and domain III (EDIII) proteins of DENV4. Western blotting analysis was used to reveal 7 mAbs (DD1-4, DD11-4, DD13-4, DD14-1, DD18-5, DD30-4, and DD31-3) that could recognize EDI-II (about 36 kDa) (Fig 2D). We also identified 2 mAbs (DD17-4 and DD27-8) that could bind to EDIII (about 17 kDa) (Fig 2E).

### Examination of neutralizing activities of mAbs

**Fig 3:**
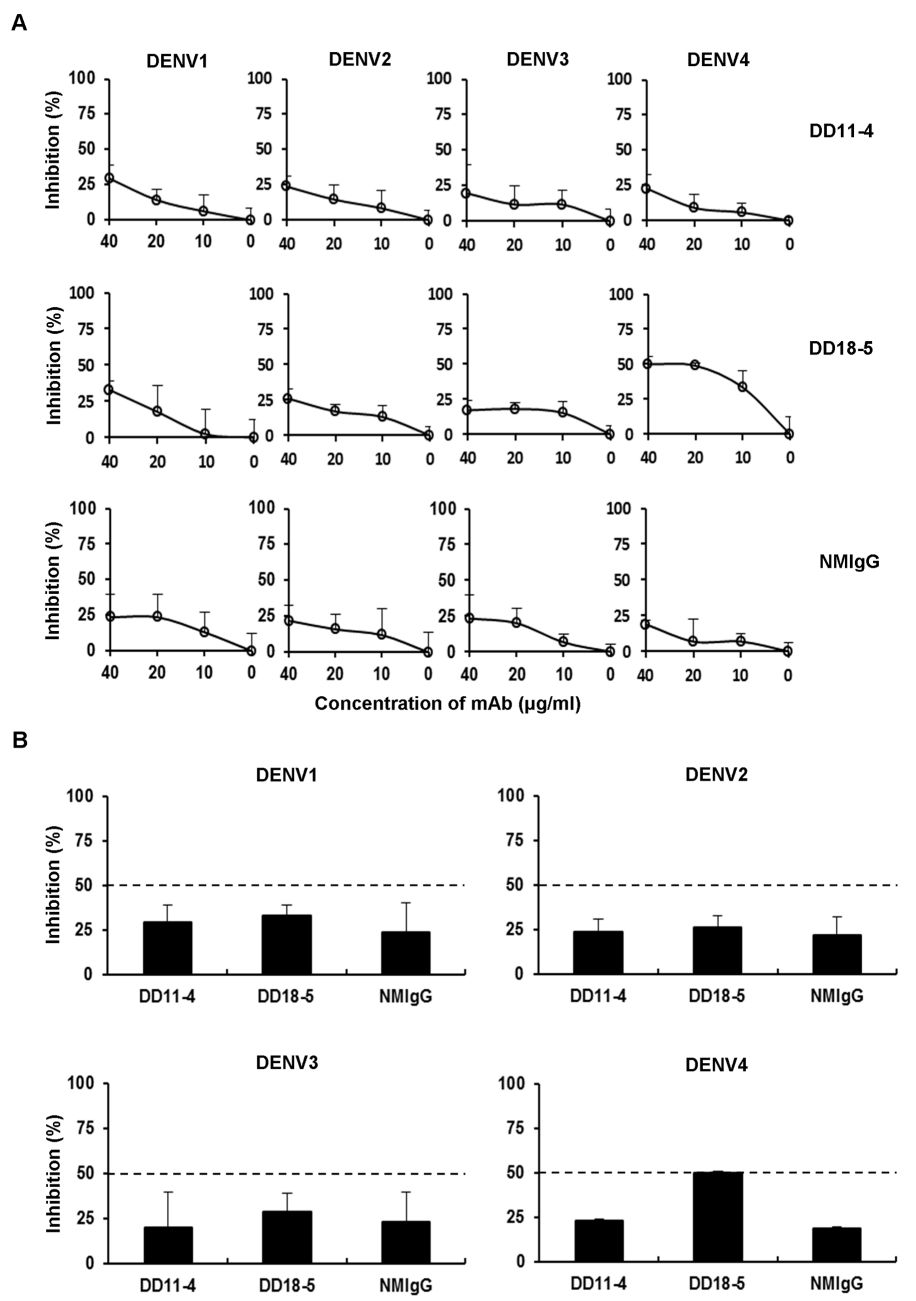
Examination of the neutralizing activity of mAbs against DENV. (A) *In vitro* neutralizing activity of mAbs against DENV1-4 was examined using the plaque reduction neutralization test (PRNT). DENV1-4 was incubated with DD11-4 or DD18-5, and used to infect BHK-21 cells. After 4 days, the cells were fixed and stained, and viral plaques were counted after washing. The inhibition percentages were shown. (B) DENV1-4 were incubated with DD11-4 or DD18-5 at 40 μg/ml, and the mixtures were then used to infect BHK-21 cells. After 4 days, viral plaques were counted and the inhibition percentages were determined. NMIgG was used as a control. Data shown are from one representative experiment of two independent experiments.

To examine the *in vitro* neutralizing activities of mAbs, we performed plaque reduction neutralization test (PRNT). Thirteen anti-E protein mAbs showed weakly neutralizing activity against DENV4 (Fig 3 and S1 Fig). DD11-4 showed non-neutralizing activity against DENV4 (PRNT_50_ titer, >40 μg/ml). DD18-5 showed sub-neutralizing activity against DENV4 (PRNT_50_ titer, ≧20 μg/ml). Furthermore, DD11-4 and DD18-5 had non-neutralizing activity against DENV1-3 (PRNT_50_ titer, >40 μg/ml). These results indicate that DD11-4 and DD18-5 are ineffective at preventing viral infection and exhibit poorly neutralizing activity against DENV4 (Table 1).

### Evaluation of enhancing activities of mAbs

**Fig 4:**
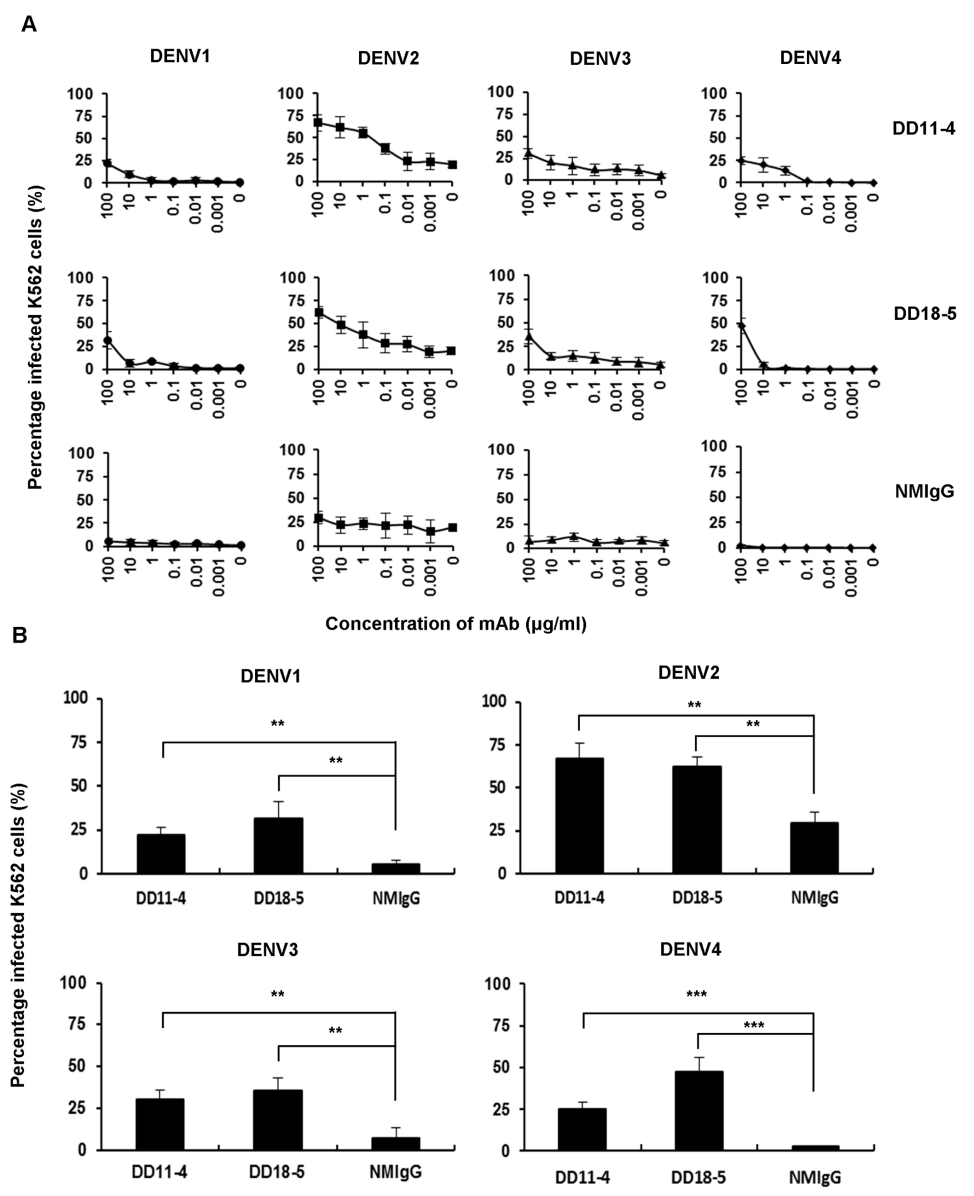
*In vitro* ADE of DENV mediated by cross-reactive mAbs. (A) K562 cells were incubated with DENV1 (MOI = 1), DENV2 (MOI = 1), DENV3 (MOI = 5), or DENV4 (MOI = 1) in the presence of the serially-diluted mAbs; the percentage of infected K562 cells was examined at 72 hours post-infection by staining with 4G2, followed by flow cytometry. (B) K562 cells were incubated with DENV1-4 and the indicated mAbs at 100 μg/ml, and the percentage of infected K562 cells was determined at 72 hours post-infection by staining with 4G2, followed by flow cytometry. NMIgG was used as a control. Data shown are from one representative experiment of two independent experiments. Unpaired Student’s *t* tests were used to calculate *P* values (**P<0.01, ***P<0.001).

The poorly neutralizing antibodies that bound to DENV1-4 are considered to have high potential for enhancing viral infection by ADE. We subsequently evaluated the *in vitro* enhancement of DENV1-4 infection by DD11-4 and DD18-5 in K562 cells, a human erythroleukemic cell line that expresses high levels of FcγRIIA [32]. DENV1-4 were incubated with serially-diluted mAbs and infected K562 cells (Fig 4A). Non-neutralizing DD11-4 or sub-neutralizing DD18-5 significantly enhanced DENV1-4 infection as compared to NMIgG, with high enhancement observed at concentration of 100 μg/ml (Fig 4B). These results indicate that DD11-4 and DD18-5 are highly potent at enhancing DENV infection.

**Fig 5:**
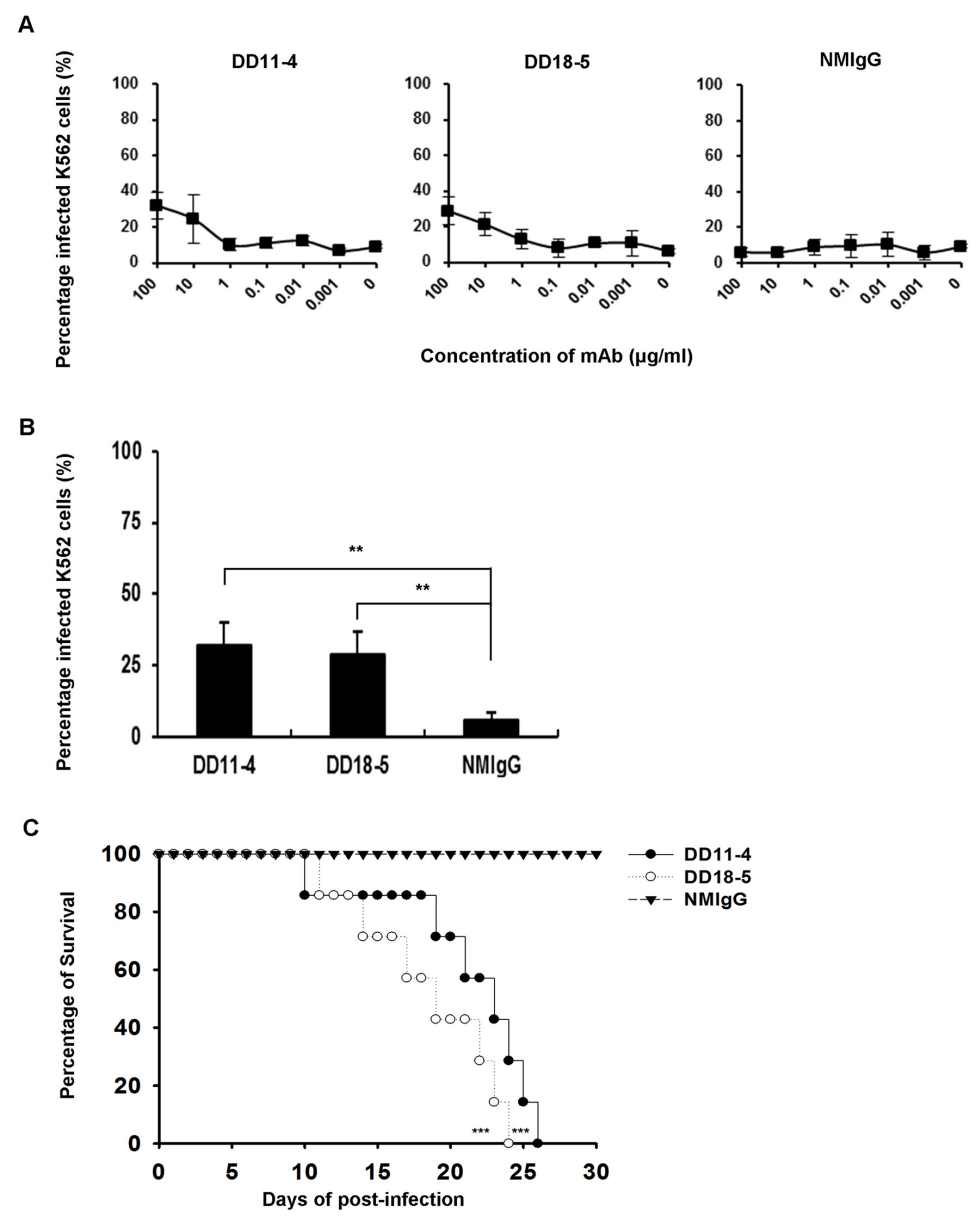
Evaluation of *in vitro* and *in vivo* ADE with cross-reactive mAbs. (A) DENV2 S221 (MOI = 5) was incubated with the serially-diluted mAbs for 1 hour at 4°C and then used to infect K562 cells. After 3 days, the cells were stained with 4G2, and analyzed by flow cytometry. (B) K562 cells were incubated with DENV2 S221 (MOI = 5) and the indicated mAbs at 100 μg/ml, and the percentage of infected K562 cells was determined at 72 hours post-infection by staining with 4G2, followed by flow cytometry. NMIgG was used as a control. Unpaired Student’s *t* tests were used to calculate *P* values (**P<0.01). (C) AG129 mice were i.v. infected on day 0 with 1 × 10^5^ pfu DENV2 S221 and i.p. treated on days -1 and 1 with 5 μg of DD11-4 (n = 7), DD18-5 (n = 7), or NMIgG (n = 8). The survival rates were recorded for 30 days. Kaplan-Meier survival curves and *P* values are shown (***P<0.001, compared to NMIgG). Data shown are from one representative experiment of two independent experiments.

The mouse-adapted DENV2 S221 was used to study enhancement of mortality in AG129 mice [29]. DENV2 S221 was incubated with serially-diluted mAbs, and infected K562 cells (Fig 5A). The results indicated that DD11-4 and DD18-5 significantly enhanced DENV2 S221 infection in K562 cells at concentration of 100 μg/ml, as compared to NMIgG (Fig 5B). Next, we confirmed the *in vivo* enhancement of mortality in AG129 mice. Mice treated with 5 μg DD11-4 or DD18-5 exhibited lethal symptoms and increased mortality as compared to control-infected mice (Fig 5C). These results suggest that DD11-4 and DD18-5 have *in vitro* enhancing activities, and are highly potent at increasing mortality in AG129 mice.

### Identification of the epitope residues

**Fig 6:**
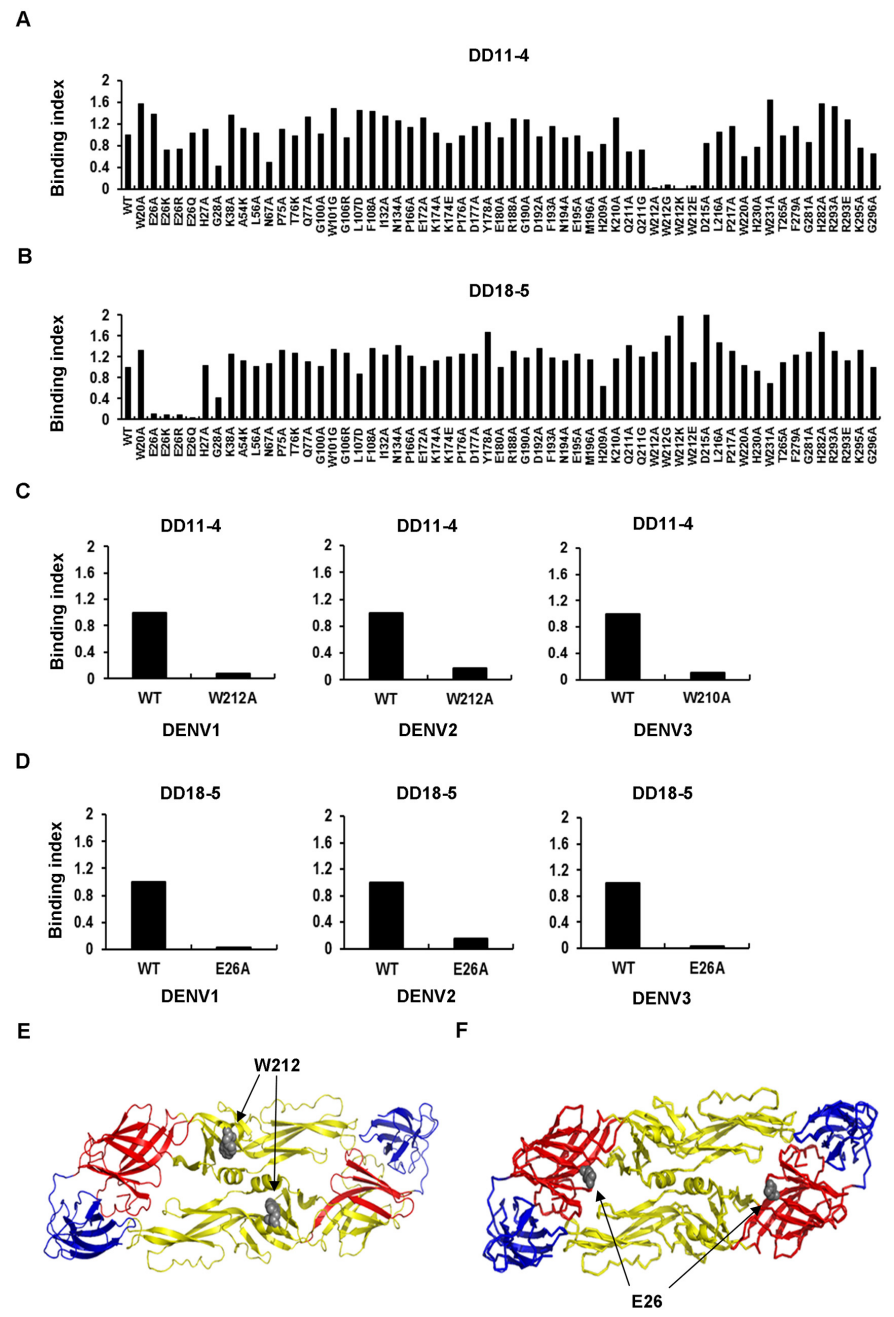
Identification of the epitope residues on E protein. BHK-21 cells were transfected with vectors expressing wild-type or mutant DENV4 E proteins. After 2 days, the cells were reacted with DD11-4 (A) or DD18-5 (B), and then analyzed by flow cytometry. The binding index of a mAb to a mutant E protein was measured. The binding indices of DD11-4 and DD18-5 were reduced by mutations at W212 and E26, respectively. Data shown are from one representative experiment of two independent experiments. (C and D) Various wild-type or mutant DENV1-3 E proteins were expressed in BHK-21 cells. After fixation and permeabilization, the cells were incubated with mAbs, and analyzed by flow cytometry. The binding index of a mAb to a mutant E protein was measured. Mutations of W212 (W210 in DENV3) and E26 led to a significant loss of binding activity of DD11-4 (C) and DD18-5 (D), respectively. (E) The epitope of DD11-4 is located at residue W212 in EDII. (F) The epitope of DD18-5 is located at residue E26 in EDI. The model is based on the DENV2 E protein model (PDB: 1OAN). EDI is shown in red, EDII is shown in yellow, and EDIII is shown in blue.

Identification of the binding epitopes of DD11-4 and DD18-5 on DENV E protein would help us delineate the mechanisms involved. Here, we used computer modeling with the Pepitope server (http://pepitope.tau.ac.il/) to predict the residues of candidate-binding sites. We then used site-directed mutagenesis to substitute the predicted residues within the constructs. After transfection and analysis by flow cytometry, the binding percentages were determined (S2 Fig). The binding indices were normalized to those of anti-EDIII mixed mAbs and calculated (Fig 6A–6D). Mutation of W212 (W210 in DENV3) on the E protein of DENV1-4 resulted in a loss-of-reactivity with DD11-4 (Fig 6A and 6C). The same methods were used to identify E26 on the E protein of DENV1-4 as the epitope residue of DD18-5 (Fig 6B and 6D). We identified that the two cross-reactive mAbs had epitopes at domain II and I, respectively, namely residue W212 for DD11-4 (Fig 6E) and residue E26 for DD18-5 (Fig 6F). These epitope residues were aligned with the amino acid sequence of the EDI-II protein of DENV1-4, revealing that W212 and E26 are conserved in DENV1-4 (Table 2).

**Table 2:** Alignment of amino acid residues recognized by DD11-4 and DD18-5 in EDI-II protein^a^.

Serotypes of DENV (strain)	Accession number (GenBank)	Amino acid of EDI-II protein	
		26^b^	212^c^
DENV4 (H241)	AAX48017	**E**	**W**
DENV4 (B5)	AAG30148	**E**	**W**
DENV4 (ThD4_0348_91)	AAU89377	**E**	**W**
DENV4 (ThD4_0087_77)	AAU89378	**E**	**W**
DENV4 (ThD4_0485_01)	AAU89379	**E**	**W**
DENV4 (ThD4_0734_00)	AAU89380	**E**	**W**
DENV4 (ThD4_0476_97)	AAU89375	**E**	**W**
DENV4 (814669)	AAK01233	**E**	**W**
DENV4 (Indonesia 1976)	AAB70680	**E**	**W**
DENV4 (TVP-376)	AGS14893	**E**	**W**
DENV4 (TVP-986)	AGS14894	**E**	**W**
DENV1 (Hawaii)	AIU47321	**E**	**W**
DENV1 (16007)	AAF59977	**E**	**W**
DENV2 (16681)	AAB58782	**E**	**W**
DENV2 (NGC)	AAA42941	**E**	**W**
DENV2 (PL046)	AHZ61501	**E**	**W**
DENV2 (PM33974)	ABO33322	**E**	**W**
DENV2 (IQT2913)	AAD32963	**E**	**W**
DENV2 (ThD2_0038_74)	ABA61185	**E**	**W**
DENV2 (ThD2_0168_79)	ABA61184	**E**	**W**
DENV2 (ThD2_0498_84)	ABA61183	**E**	**W**
DENV2 (Jamaica/N.1409)	AAA42942	**E**	**W**
DENV2 (TSV01)	AAK67712	**E**	**W**
DENV2 (Tonga/74)	AAV70829	**E**	**W**
DENV2 (I348600)	AAW31413	**E**	**W**
DENV3 (ThD3_0183_85)	AAV88402	**E**	**W** ^d^
DENV3 (H87)	AAA99437	**E**	**W** ^d^

^a^ The EDI-II protein sequences of strains of DENV1-4 were aligned. Single letter amino acid abbreviations are shown.

^b^ The epitope residue of DD18-5 is shown in boldface type.

^c^ The epitope residue of DD11-4 is shown in boldface type.

^d^ The epitope residue of DD11-4 is W210 in DENV3, and is shown in boldface type.

## Discussion

Monoclonal antibodies (mAbs) are broadly used for viral diagnosis, pathological research, and treatment of infectious diseases. In this study, we generated sixteen novel mAbs against DENV4. We observed that ten of these mAbs were serotype-specific, and six were cross-reactive. Western blotting was used to show that most of the mAbs recognize EDI-II of DENV4 E protein. The cross-reactive DD11-4 and DD18-5 mAbs, which recognize EDI-II, have poor neutralizing activities and enhance DENV1-4 infection in K562 cells. Furthermore, DD11-4 and DD18-5 increased mortality in AG129 mice. Using structure prediction and epitope mapping, we identified the epitope residues of DD11-4 and DD18-5. These findings will be useful for investigating the mechanisms of dengue infection.

The antibodies against E protein play important roles in neutralization and regulation of DENV infection [15–18]. The E protein monomer consists of three domains (EDI, EDII, and EDIII). Previous studies have shown that serotype-specific mouse mAbs against EDIII have neutralizing activity [18, 23, 24]; however, few antibodies bind to this region in human [33]. Recently, it was shown that human neutralizing antibodies target complex epitopes on dengue particles [34]. However, a large fraction of cross-reactive and weakly neutralizing human antibodies against E or prM proteins can enhance viral infection through ADE [25, 35]. Thus, identification of B-cell epitopes of DENV E protein, which induce cross-reactive and poorly neutralizing antibodies, is an important step toward combating ADE. In this study, analysis of binding domains by Western blotting revealed that seven mAbs recognize EDI-II, and two mAbs recognize EDIII. The other four anti-E protein mAbs could not bind to EDI-II or EDIII. These mAbs may recognize conformational epitopes of E protein. Next, PRNT was used to examine the *in vitro* neutralizing activities of anti-E mAbs. Seven anti-EDI-II mAbs, two anti-EDIII mAbs, and four anti-E mAbs showed poorly neutralizing activity against DENV4 (Table 1). In addition, DD11-4 and DD18-5 are poorly neutralizing against DENV1-4 (Fig 3). These results indicate that anti-EDI-II DD11-4 and DD18-5 are cross-reactive mAbs against DENV1-4, with weak neutralizing activities.

The exacerbation of dengue symptoms during secondary infections has been proposed to be caused by ADE. This process enhances infection by a heterologous serotype of DENV [36]. The enhancement of DENV infection by antibodies can either be tested *in vitro* with Fcγ receptor (FcγR)-expressing cells or examined *in vivo* in AG129 mice [5, 26, 37]. Until now, the interaction between DENV pathogenesis and antibodies was not well understood. K562 cells express FcγRIIA, and thus can be used to evaluate *in vitro* enhancing activities. Our results indicate that DD11-4 and DD18-5 enhance viral infection over a broad range of concentrations (Figs 4A and 5A). Furthermore, DD11-4 and DD18-5 enhanced viral infection at high concentrations (Figs 4B and 5B), compared to NMIgG. Mouse-adapted DENV2 S221 was previously used to study dengue disease via ADE in AG129 mice [29]. Here, we further demonstrated that mortality in AG129 mice is enhanced by DD11-4 and DD18-5 (Fig 5C). These results indicate that DD11-4 and DD18-5 have *in vitro* and *in vivo* enhancing activity.

Identification of the epitopes of mAbs helps us to understand the mechanisms of dengue infection. Combining computational structure analysis [38] with VLP mutants [18] is a useful approach for identifying B-cell epitopes. Based on the *in silico* predictions, we subjected VLP mutants to a binding assay as a method of screening for B-cell epitopes. DD11-4 and DD18-5 recognized amino acid residues in domain II (W212) and domain I (E26), respectively. These amino acid residues were first reported to be involved in weak neutralization of DENV infection and ADE. Previous studies showed that G106 and L107 are the epitope residues of enhancing mAb 4G2 [11]. In this study, we also verified that DD11-4 and DD18-5 do not bind to G106 and L107 in the fusion loop. These findings suggest that DD11-4/DD18-5 and 4G2 enhance DENV infection by different mechanisms. Besides, mutations at W212 may change in E protein structure and affect the binding of mixed anti-EDIII mAbs (S2 Fig). Cryo-electron microscopy (cryo-EM) may be a viable approach for investigating epitope residues that change the conformation of E proteins through binding of enhancing mAbs.

The E protein is regarded as an important target for dengue vaccines [39]. However, the efficacy of such vaccines and the effect of substituting enhancing epitopes on safety are yet to be fully examined. Previous reports indicated that DNA vaccine candidates containing substitutions in the fusion loop (at G106 and L107) and the cross-reactive epitopes of EDIII (at K310, E311, and P364) provide protective immune responses against DENV1 or DENV2 [40, 41]. In addition, the substituted DNA vaccine (at N8) improves immunogenicity against DENV2 [42]. In this study, we have identified new epitope residues of enhancing antibodies, and we believe that substitution of these enhancing epitope residues and subsequent evaluation of the effect on ADE will be of interest to vaccine development.

In conclusion, we generated sixteen novel mAbs which recognize different target proteins in DENV4. The cross-reactive DD11-4 and DD18-5 mAbs against EDI-II were found to have poor neutralizing activity, but high enhancing activity. Although the relationship between ADE and severe illness is still unclear, our mAbs may be useful tools for studying this interaction. Identification of the enhancing epitopes has provided valuable information for the development of therapies against DENV infection.

## Supporting Information

S1 Fig Examination of the neutralizing activity of mAbs against DENV4. *In vitro* neutralizing activity of mAbs against DENV4 was examined using the plaque reduction neutralization test (PRNT), as described in the Materials and Methods. The indicated mAbs at 40 μg/ml were incubated with DENV4. Then, the mixtures were used to infect BHK-21 cells. After 4 days, viral plaques were counted and the inhibition percentages were determined.
(TIF)

S2 Fig Determination of the epitope residues of DD11-4 and DD18-5 on E protein. The wild-type or mutant DENV1-4 E proteins were expressed in BHK-21 cells. After fixation and permeabilization, the collected cells were incubated with DD11-4 (A and C), DD18-5 (B and D), or mixed mAbs. The binding percentages were analyzed by flow cytometry. Substitution of W212 (W210 in DENV3) led to a significant loss of binding activity of DD11-4 (A and C). Substitution of E26 led to a significant loss of binding activity of DD18-5 (B and D). Data shown are from one representative experiment of two independent experiments.
(TIF)
