# Supplementary figures and images for: Generation of Monoclonal Antibodies against Dengue Virus Type 4 and Identification of Enhancing Epitopes on Envelope Protein
Source: PLoS One. 2015 Aug 26;10(8):e0136328. doi: 10.1371/journal.pone.0136328 (PMC4550467; doi:10.1371/journal.pone.0136328)

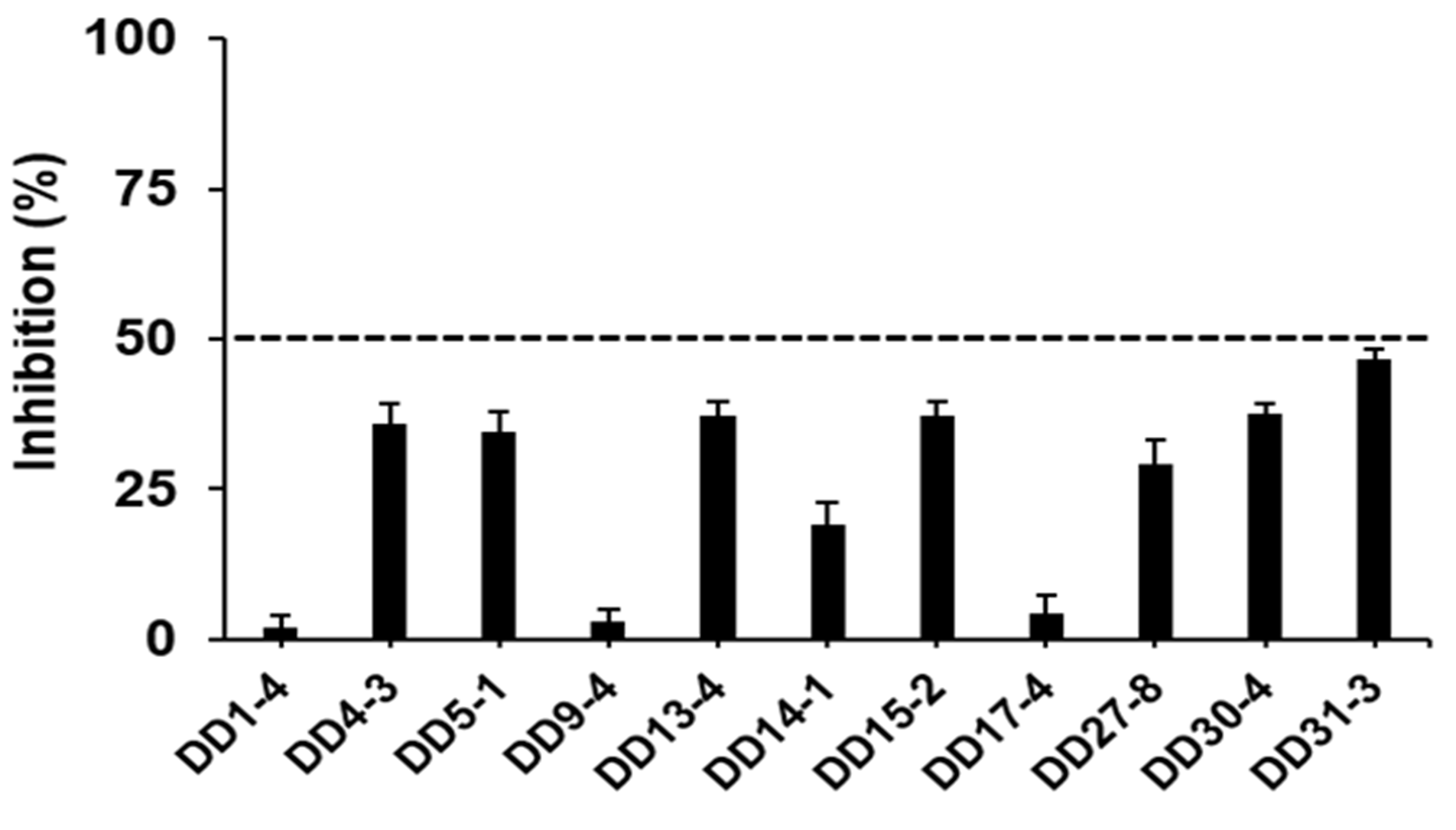

Supplement: S1 Fig — In vitro neutralizing activity of mAbs against DENV4 was examined using the plaque reduction neutralization test (PRNT), as described in the Materials and Methods. The indicated mAbs at 40 μg/ml were incubated with DENV4. Then, the mixtures were used to infect BHK-21 cells. After 4 days, viral plaques were counted and the inhibition percentages were determined. (TIF) [file pone.0136328.s001.tif]

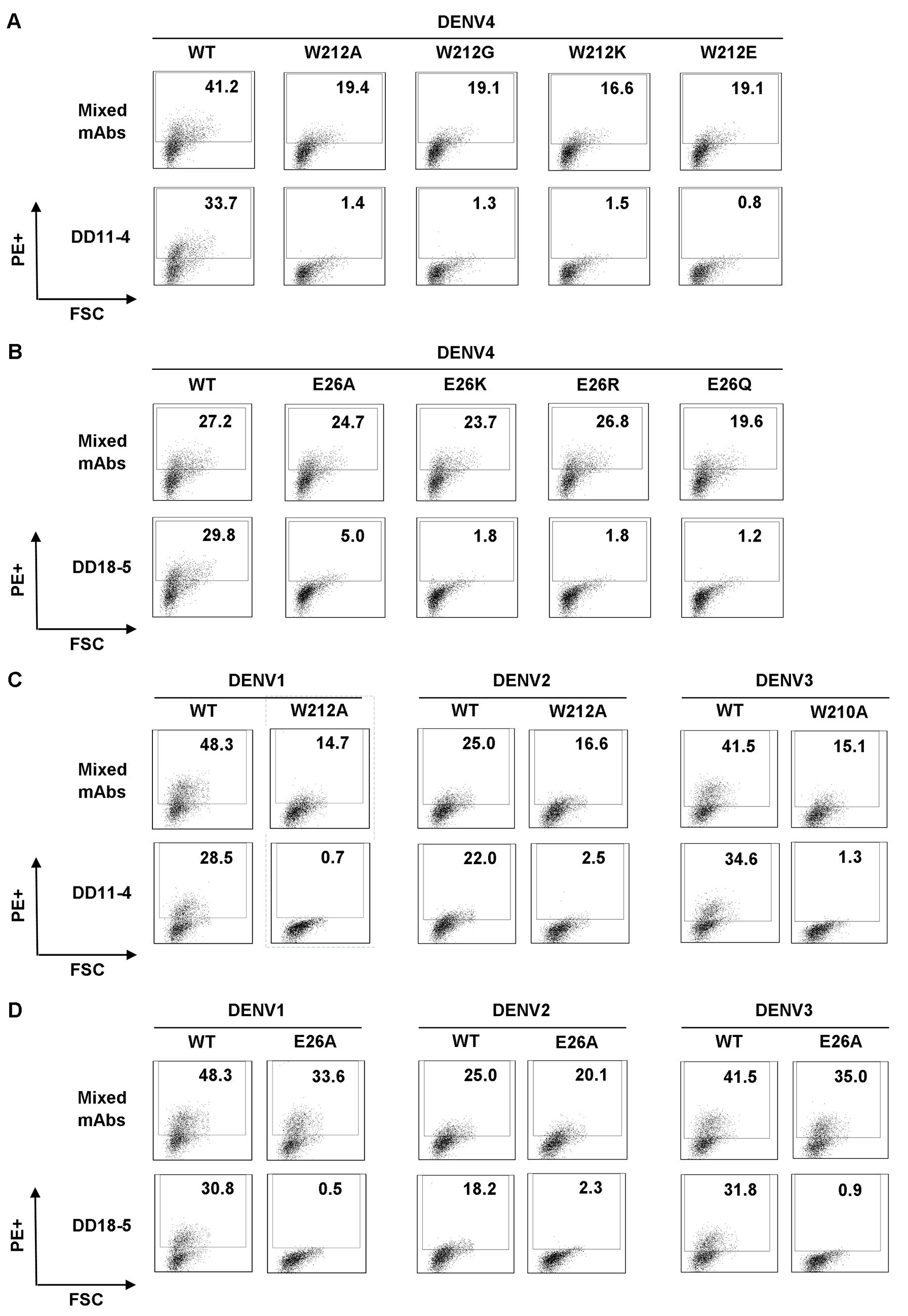

Supplement: S2 Fig — The wild-type or mutant DENV1-4 E proteins were expressed in BHK-21 cells. After fixation and permeabilization, the collected cells were incubated with DD11-4 (A and C), DD18-5 (B and D), or mixed mAbs. The binding percentages were analyzed by flow cytometry. Substitution of W212 (W210 in DENV3) led to a significant loss of binding activity of DD11-4 (A and C). Substitution of E26 led to a significant loss of binding activity of DD18-5 (B and D). Data shown are from one representative experiment of two independent experiments. (TIF) [file pone.0136328.s002.tif]
